# A Comprehensive Assessment of the Associations Between Season of Conception and Birth Defects, Texas, 1999–2015

**DOI:** 10.3390/ijerph17197120

**Published:** 2020-09-29

**Authors:** Elisa Benavides, Philip J. Lupo, Peter H. Langlois, Jeremy M. Schraw

**Affiliations:** 1 Center for Epidemiology and Population Health, Department of Pediatrics, Baylor College of Medicine, Houston, TX 77030, USA; Elisa.Benavides@bcm.edu (E.B.); Philip.Lupo@bcm.edu (P.J.L.); 2 Section of Hematology-Oncology, Department of Pediatrics, Baylor College of Medicine, Houston, TX 77030, USA; 3 Division of Epidemiology, Human Genetics, and Environmental Sciences, University of Texas School of Public Health, Austin, TX 78701, USA; Peter.Langlois@uth.tmc.edu

**Keywords:** birth defects, congenital defects, seasonal variation, epidemiology, season of conception, phenome-wide association study

## Abstract

Birth defects prevalence may vary seasonally, but previous studies have focused on a few commonly occurring phenotypes. We performed a phenome-wide association study (PheWAS) in order to evaluate the associations between season of conception and a broad range of birth defects. Date of conception was estimated for all livebirths and birth defect cases in Texas from 1999–2015 using data from vital records, provided by the Texas Department of State Health Services Center for Health Statistics. Birth defects diagnoses were obtained from the Texas Birth Defects Registry, a statewide, active surveillance system. We estimated prevalence ratios (PRs) for phenotypes with ≥50 cases according to conception in spring (March-May), summer (June–August) or fall (September–November) relative to winter (December–February), using Poisson regression. Season of conception was associated with 5% of birth defects studied in models adjusted for maternal age, education, race/ethnicity, and number of previous livebirths. Specifically, summer conception was associated with any monitored birth defect (PR 1.03, 95% CI 1.02–1.04) and five specific phenotypes, most notably Hirschsprung disease (PR 1.46, 95% CI 1.22–1.75). These findings suggest that seasonally variable exposures influence the development of several birth defects and may assist in identifying novel environmental risk factors.

## 1. Introduction

Birth defects are health problems or abnormal physical changes that can affect any part of the body (e.g., brain, heart, and intestines). Relatively common birth defects include structural defects such as neural tube defects and congenital heart disease, as well as chromosomal anomalies such as trisomy 21 (i.e., Down syndrome) [1]. Birth defects can be identified at various times, including: during pregnancy through tests such as amniocentesis, echocardiograms, and ultrasounds; at birth; or any time after birth [2,3]. In the United States (US), birth defects affect approximately 3% of births each year and account for 20% of infant deaths [1]. Those who do survive often have life-long medical issues that may impact quality of life. In spite of their prevalence and clinical significance, approximately 60% of these conditions are of unknown etiology [4]. Several environmental exposures have been demonstrated to cause a spectrum of birth defects, including antiepileptic drugs and pregestational diabetes [5,6,7,8,9]. One “exposure” that has been evaluated in relation to the risk of birth defects is season of conception [10,11,12].

As maternal diet, exposure to infectious agents, and ambient temperature vary throughout the year, season of conception can serve as a proxy for these factors and could provide new insights into the etiologies of birth defects overall, as well as specific phenotypes. A handful of studies have demonstrated that birth defects, including congenital heart disease, cleft lip and palate, and lower limb reductions [10,11,12], significantly vary by season of conception. In spite of these associations, there have been few efforts to systematically evaluate the role of this factor on birth defects overall, or among less frequent phenotypes.

Population-based birth defect registries ascertain the occurrence of birth defects in a defined geographic area for all pregnancies that last longer than a specific duration (e.g., ≥20 weeks of gestation). Importantly, these data can be linked with demographic and exposure data to evaluate associations between these factors and the prevalence of birth defects [13]. Using these data, our objective was to systematically characterize the associations between season of conception and birth defects recorded by the Texas Birth Defects Registry (TBDR) with a phenome-wide association study (pheWAS) methodology. The pheWAS methodology was designed to study pleiotropic associations between a single gene, locus, or variant across multiple phenotypes in genetic epidemiology studies [14,15]. However, this approach has recently been extended to evaluate associations between maternal exposures and birth defects [16]. In spite of the robust nature of this approach, to our knowledge, this is the first pheWAS to evaluate the association between season of conception and a range of birth defects.

## 2. Methods

### 2.1. Birth Defect Ascertainment and Classification

Our analysis was focused on all infants and fetuses with a definite diagnosis of any structural or chromosomal birth defect, regardless of pregnancy outcome that was recorded by the TBDR from deliveries in 1 January 1999 through 31 December 2015. The TBDR is a population-based, active surveillance system that ascertains cases from multiple sources, including birth and fetal death certificates, and records from hospitals and other facilities where affected children are born or treated. This information is abstracted into case records, which are subject to review by registry staff. TBDR records are linked to birth certificates and fetal death certificates, resulting in >95% of cases being linked to their vital record. Diagnoses flagged as “possible” or “probable” based on qualifiers found in the medical record (approximately 4%) were excluded from our analyses. The TBDR classifies birth defects using Centers for Disease Control (CDC)-modified British Paediatric Association (BPA) 6-digit codes. The original 6-digit codes were collapsed to the first four digits for comparability to the International Classification of Disease, Ninth Revision (ICD-9). These are referred to as BPA4 codes. Exceptions to this include: spina bifida (collapsed to the first three digits to combine spina bifida with and without hydrocephaly); cleft lip alone (749.1) and cleft lip with cleft palate (749.2), which were both assigned 749.1, and cleft lip with or without cleft palate; omphalocele (756.700) which was assigned 756.70; and gastroschisis (756.710), which was assigned 756.71. In addition, a BPA4 code of 888.8 was created to reflect the presence of any monitored birth defect in a case. Cases with two or more of the same BPA4 code were de-duplicated to ensure that they had a maximum of one of each code. All livebirths in Texas for the same time period between 1 January 1999 and 31 December 2015 are included in this study for birth prevalence denominators.

From birth and fetal death certificates, data was obtained on infant’s sex, maternal age, maternal race/ethnicity, maternal education, number of previous livebirths, and estimated date of conception (EDC). Maternal age was grouped into six categories (<20, 20–24, 25–29, 30–34, 35–39, and ≥40 years). Maternal race/ethnicity was classified as Hispanic, non-Hispanic White, non-Hispanic Black, and other. Maternal education was classified as less than high school, high school, or greater than high school and number of previous livebirths were grouped into four categories (0, 1, 2, ≥3). EDC was calculated based on last menstrual period (LMP) and the clinical estimate of gestational age. If both LMP and estimate of gestational age were present, LMP took precedence, and if neither variable was available, EDC was set to missing.

This study was approved by the Baylor College of Medicine and Texas Department of State Health Services Institutional Review Boards (IRB), IRB numbers H-31777 and 18-046, respectively, and performed in accordance with the principles of the Declaration of Helsinki. Strengthening the Reporting of Observational Studies in Epidemiology (STROBE) guidelines [17] were followed.

### 2.2. Statistical Analysis

The distributions of maternal characteristics among cases and livebirths were summarized using counts and percentages. A season of conception (winter, spring, summer, and fall) variable based on the EDC and on the temperature cycle was created. We adhered to meteorological and climatological recommendations and classified December, January, and February as winter months; March, April, and May as spring months; June, July, and August as summer months; and September, October, and November as fall months [18]. Birth prevalence per 10,000 livebirths was estimated for each birth defect among the offspring conceived in each season. In addition, birth prevalence for any monitored birth defect by month of conception was estimated.

Only those BPA4-coded structural birth defects (i.e., all diagnoses derived from CDC-BPA codes 740.000-759.000) with ≥50 total cases were included in the PheWAS. We computed crude and multivariable Poisson models to estimate the prevalence ratio (PR) and 95% confidence interval (CI) for the index birth defect among offspring by season of conception as a categorical variable, with winter as the referent. We additionally computed models for any monitored birth defect in offspring based on month of conception, with January as the referent. All multivariable models were adjusted for maternal age, race/ethnicity, education, and number of previous livebirths.

**Figure 1:**
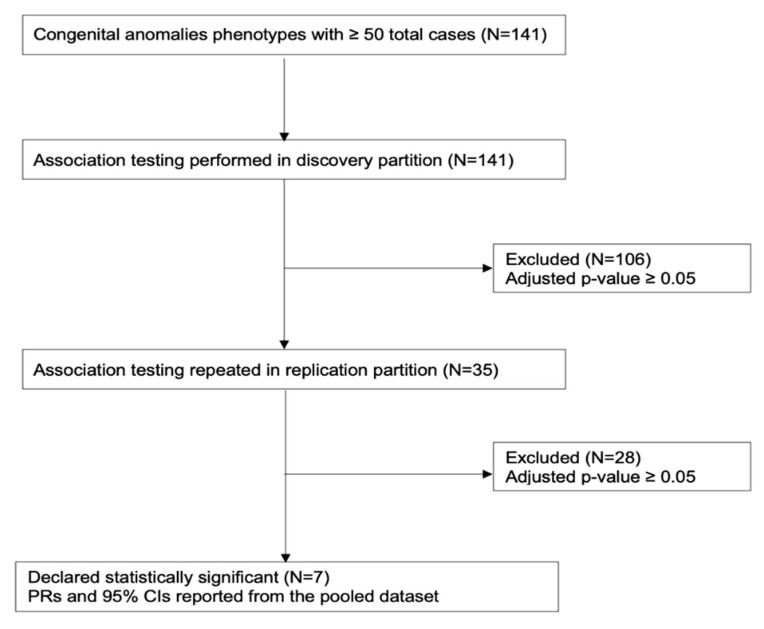
Overview of association testing methodology and phenotypes included at each stage of analysis.

Figure 1 provides an overview of the association testing methodology and criteria for declaring statistical significance. Before beginning analysis, the data were randomly divided into discovery (60% of births) and replication (the remaining 40% of births) partitions. The models were first computed in the discovery partition. Originally, we evaluated statistical significance using a Bonferroni-adjusted threshold of *p* < 3.55 × 10^−4^. This was derived by dividing the desired family-wise error rate of α = 0.05 by the number of association tests (*n* = 141) to be performed. While season of conception was associated with the prevalence of any monitored birth defect at this threshold, no individual birth defect was significant. Consequently, we evaluated their statistical significance at a nominal *p*-value of 0.05. The two stage modeling approach reduces the possibility of Type I errors, as an association observed in the discovery partition must then be independently validated in the replication partition. Birth defects that were associated with ≥1 season of conception in the discovery partition were validated in the replication partition. A season-anomaly association was declared statistically significant if they were associated at *p* < 0.05 in multivariable models and showed the same direction of effect in both partitions. For these birth defects, we present PRs and 95% CIs estimated in the pooled dataset. Analyses were performed in R v3.6.2 (R Foundation for Statistical Computing, Vienna, Austria).

## 3. Results

During the period of 1999–2015, there were 6,543,397 livebirths and 964,054 birth defects diagnosed. Table 1 shows maternal demographic characteristics among livebirths and cases with birth defects. Among all livebirths and birth defects cases, respectively, the distribution of season of conception was as follows: winter 1,686,752 (25.8%) and 77,604 (25.5%), spring 1,634,191 (24%) and 75,312 (24.7%), summer 1,565,866 (23.9%) and 74,181 (24.4%), and fall 1,642,220 (25.1%) and 76,733 (25.2%).

### 3.1. Prevalence of Any Monitored Birth Defect According to Month of Conception

**Figure 2:**
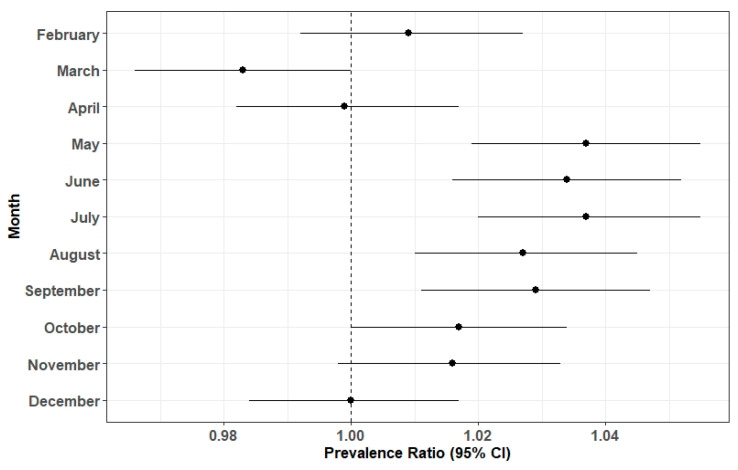
Adjusted Prevalence Ratios (PRs) of any monitored birth defect in the overall dataset by month of conception, with January as the referent. PRs adjusted for maternal age, race/ethnicity, education, and number of previous livebirths.

Figure 2 presents adjusted PRs for any monitored birth defect in the overall dataset by month. There were significantly increased PRs for any monitored birth defect among conceptions in May, June, July, August, and September, relative to January. Conception in March was negatively associated with prevalence of any birth defect compared to January. These results supported that our classification of seasons captured the monthly variation in the prevalence of birth defects.

### 3.2. Prevalence of Specific Birth Defects According to Season of Conception

**Figure 3:**
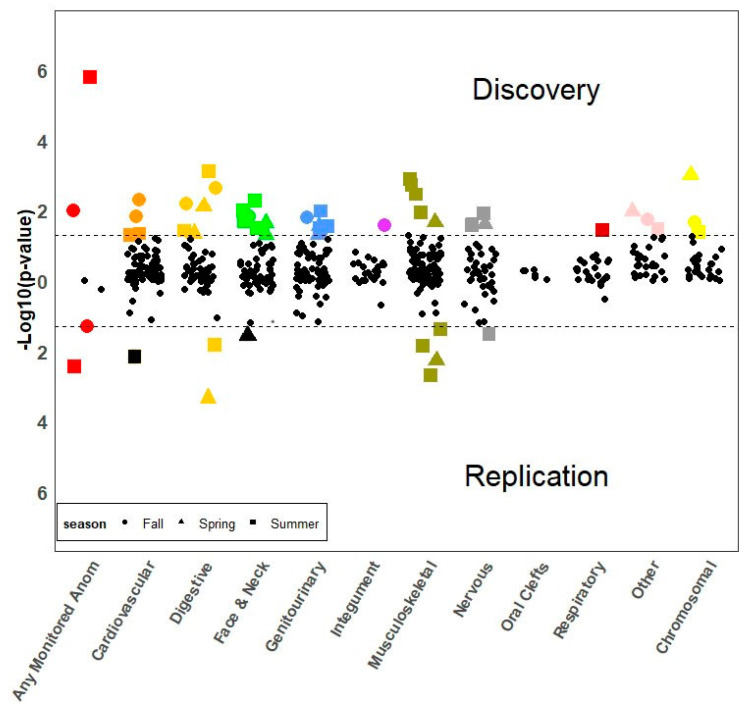
Associations between season of conception and birth defects. Birth defects associated with season of conception at *p* < 0.05 in discovery (upper panel) (*n* = 35) were re-tested in replication (lower panel). Those associated with season of conception in replication with the same direction of effect and at *p* < 0.05 were declared significant (*n* = 7 unique birth defects and 9 season-birth defect associations).

One hundred and forty one phenotypes with ≥50 total cases were included in the PheWAS. This represented 140 unique birth defects as well as any monitored birth defect. In the discovery partition, season of conception was associated with any monitored birth defect at *p* < 3.55 × 10^−4^ and with 34 specific birth defects at *p* < 0.05 (Figure 3, upper panel, and Supplemental Table S1). Increased PRs were observed for birth defects involving the nervous, cardiovascular, digestive, genitourinary, musculoskeletal, and integumentary systems. Of these 35 phenotypes, some such as common truncus and “other anomalies of the intestine” (e.g., microcolon), were significantly associated with more than one season of conception, resulting in a total of 42 season-anomaly associations. Notably, 18 of 42 season-anomaly associations (42.9%) involved increased prevalence for children conceived during the summer, ranging from PRs of 1.03 for any monitored birth defect to 2.07 for unspecified chromosomal abnormalities.

Seven of these 35 candidate phenotypes, including any monitored birth defect, were also associated with the same season of conception in the same direction and at *p* < 0.05 in the replication partition (Figure 3, lower panel, and Supplemental Table S2). Those that replicated included: varus deformities of the feet; other anomalies of the lower limbs such as brachydactyly, rocker-bottom foot, and fusion of the sacroiliac joint; congenital hypertrophic pyloric stenosis; reduction anomalies of the brain including holoprosencephaly; Hirschsprung disease (congenital megacolon) and other congenital functional disorders of the colon such as total intestinal aganglionosis; and anomalies of the skull and face bones. Any monitored birth defect and anomalies of the skull and face bones replicated in more than one season, resulting in nine season-birth defect associations. Offspring conceived in the spring were more likely to be diagnosed with anomalies of the skull and face bones (PR 1.10, 95% CI 1.03–1.18), and offspring conceived in the fall were more likely to be diagnosed with any monitored birth defect (PR 1.02, 95% CI 1.00–1.03). The remaining season-birth defect associations involved summer conceptions, with increased prevalence relative to winter conceptions. The strongest association was observed for Hirschsprung disease and other congenital functional disorders of the colon (PR 1.42, 95% CI 1.07–1.89) for children conceived in the summer months relative to the winter months.

To maximize precision, PRs and 95% CIs were calculated for birth defects that replicated in the pooled dataset. Table 2 presents adjusted PRs and 95% CIs for the seven specific replicated season-birth defect associations. There was a greater prevalence of any monitored birth defect among offspring conceived in the summer (PR 1.03, 95% CI 1.02–1.04) or fall (PR 1.02, 95% CI 1.01, 1.03). Similar to the replication partition, the strongest association was observed for Hirschsprung disease/other congenital functional disorders of the colon among children conceived in the summer (PR 1.46, 95% CI 1.22–1.75). Among fall conceptions, the strongest association was observed for anomalies of skull and face bones (PR 1.05, 95% CI 1.01–1.10). The only birth defect for which a difference in prevalence among children conceived in the spring was observed was for congenital hypertrophic pyloric stenosis (PR 0.89, 95% CI 0.84–0.94).

## 4. Discussion

We present the associations between season of conception and a broad range of birth defects, estimated in an unselected population of all livebirths and birth defect cases from Texas for the period of 1999–2015. Using a stringent analytic approach, 5% of birth defects were associated with season of conception. Because previous studies have focused on more common birth defects, such as cleft lip and palate, cardiac anomalies, and neural tube defects (NTDs), scant data are available for many anomalies [12,19,20,21]. Our findings provide evidence of increased prevalence of several such birth defects among offspring conceived in the summer relative to the winter. Understanding how season of conception is associated with birth defects can assist in identifying risk factors and guiding public health practices to educate women in reproductive planning.

A number of epidemiological studies have investigated the role of seasonal variation in the prevalence of birth defects. However, these studies have largely focused on a few common birth defects. One of the largest studies to date investigated 42 birth defects across body systems by season of conception using four different statistical tests [10]. Specifically, Caton observed a 6-month peak seasonal variability period in 17 birth defects, including Hirschsprung disease (peak April–September) and lower limb reduction deformities (peak March–August) among children born in New York between 1992 and 2006 [10]. Conversely, Luteijn et al. studied birth defects from 20 European birth defect registries for births conceived from 2000–2008 and found the peak prevalence of lower limb reduction deformities in children conceived in January [11]. Even though 3-month increments were used in this study, we observed increased prevalence of Hirschsprung disease and other congenital functional disorders of the colon in the summer (June–August), consistent with the peak period identified by Caton [10]. Associations between varus deformities of the feet and “other anomalies of the lower limb” and conception in the summer were observed, again consistent with prior studies. Caton [10] did not find a significant seasonal trend in pyloric stenosis, whereas we observed a lower prevalence among spring conceptions. Likewise, to our knowledge, this is the first study to report an association between season of conception and anomalies of the skull and face bones.

Siffel et al. examined the prevalence of nine birth defect groups, including anencephaly, spina bifida, total NTDs, cleft palate, and anomalies of the pulmonary and aortic valve, among children born to residents of Atlanta from 1978–2001 [22]. The authors found seasonal peaks for anencephaly (March–August), anomalies of the pulmonary valve (September) and anomalies of the aortic valve (July) [22]. In contrast, in this study, seasonal variation in the prevalence of congenital heart disease was not observed. However, it is important to note the methodological distinctions in these analyses. Siffel et al. [22] evaluated month of birth rather than month of conception. Month of birth may be the same for two children of different gestational ages, who would have been conceived at different times and may have different in utero exposures. Therefore, time of conception may be a more sensitive marker for in utero exposures than month of birth. Caton and Siffel et al. both applied the Hewitt–Rogerson test and the Walter–Elwood test to analyze variation and establish seasonality [10,22]. The Hewitt–Rogerson test is a nonparametric test that sums all possible sequences of consecutive six months increments and identifies the 6-month period with the maximum rank sum [23]. The Walter–Elwood test is a parametric test that tests the amplitude of seasonal variation [24]. In this study, Poisson regression was used to estimate the relationship between season of conception and individual birth defects.

This study found significantly increased prevalence of birth defects for children conceived in the spring and summer. In Texas, temperatures typically reach 90° F in May and can exceed 100° F by August [25]. High temperatures can increase ozone concentrations and trap pollutants closer to the ground [26]. Animal studies indicate that maternal heat exposure during early pregnancy may induce fetal cell death or malformations [19,27]. In support of this, Lin et al. found increased odds of congenital heart disease for offspring of women who experienced more frequent or longer lasting extreme heat events during the spring or summer [20]. Stingone et al. studied the associations between ventricular septal defects (VSD) and fine particulate matter (PM) exposure in early pregnancy [26]. The authors found that offspring of women exposed to an extreme heat event with high levels of fine PM, had 1.59 (95% CI 0.94, 2.71) times the odds of having perimembranous VSD compared to women exposed to an extreme heat event with low exposure to fine PM [26]. Bekkar et al. conducted a systematic review on air pollution and heat exposure on adverse pregnancy outcomes, such as preterm birth, stillbirth, and low birth weight [28]. Although the authors did not conduct a meta-analysis due to heterogeneity of study populations across geographic locations, 49 of the 58 (84%) articles that focused on air pollutants (PM and ozone) and 9 of the 10 articles that focused on heat showed significant direct associations with an adverse pregnancy outcome. Our findings suggest that it is important to further investigate the associations of air pollution and extreme heat exposure with birth defects.

There are additional seasonal exposures that may contribute to birth defects. For example, there is some evidence that maternal diet and exposure to infectious agents vary seasonally [11,29,30,31]. Such exposures may explain the increase in prevalence of certain birth defects in children conceived during seasons that typically are not associated with high temperatures. In addition, social determinants of health (e.g., occupation, transportation, and access to support services) may interact with seasonal exposures to influence birth defects risk. Therefore, further epidemiological studies should be conducted to identify seasonal exposures that are related to specific birth defects more precisely.

This study has several key strengths. The large sample size allowed us to investigate the associations between season of conception and a broad range of individual birth defects, which has not been possible in previous studies. Because analyses were performed in an unselected, racially, and ethnically diverse population of Texas women, we anticipate that our findings will be generalizable to the general US population. Another strength is that season of conception was evaluated rather than season of birth. The early weeks of pregnancy are a critical developmental period and the use of birth month may lead to misclassification as it does not account for gestational length [10].

A limitation of this study was the use of vital record data for estimating date of conception using LMP and clinical estimate of gestational age. We did not directly measure certain exposures that we hypothesize may underlie seasonal variation in the prevalence of birth defects (e.g., extreme heat). This may lead to non-differential misclassification and could bias our results towards the null. More comprehensive assessment of these exposures may result in the detection of stronger associations. Additionally, information not ascertained as part of birth records (e.g., social determinants of health, family history of birth defects) could not be adequately accounted for in our assessment. Finally, another limitation of the study is that we aggregated some related birth defects based on BPA4 prefixes, for example, cleft lip alone and cleft lip with cleft palate. This could obscure patterns that are specific to one or the other. However, it is justifiable given that, in many cases, these phenotypes likely have shared etiologies.

## 5. Conclusions

Our findings suggest that a number of birth defects are associated with the season of conception. These findings offer clues to critical developmental periods and exposures (e.g., extreme heat and PM, infectious disease). This argues for additional research into putative teratogens that vary by season. As this study is one of the first to investigate a comprehensive range of specific birth defects, it is an important advance in our understanding of how timing of conception is associated with birth defects. Our findings may help inform the search for novel risk factors as well as prevention and surveillance efforts.

## Figures and Tables

**Table 1:** Demographic characteristics of livebirths and birth defects cases in Texas, 1999–2015.

	All Livebirths, N (%)	Birth Defects Cases, N (%)
**Maternal age (years)**		
10–19	831,365 (12.7)	36,245 (11.9)
20–24	1,769,279 (27.0)	77,171 (25.3)
25–29	1,784,385 (27.3)	79,903 (26.2)
30–34	1,387,169 (21.2)	66,081 (21.7)
35–39	632,649 (9.7)	35,202 (11.6)
≥40	138,010 (2.1)	9997 (3.3)
**Maternal race/ethnicity**		
Hispanic	3,165,219 (48.4)	144,452 (47.5)
Non-Hispanic white	2,328,963 (35.6)	112,837 (37.1)
Non-Hispanic black	738,796 (11.3)	33,733 (11.1)
Other	302,756 (4.6)	13,385 (4.4)
**Maternal education**		
<High school	1,841,020 (28.3)	85,824 (28.8)
High school	1,809,367 (27.8)	79,841 (26.8)
>High school	2,849,877 (43.8)	132,471 (44.4)
**Previous livebirths**		
0	2,490,880 (38.6)	120,918 (40.8)
1	2,007,439 (31.1)	86,992 (29.3)
2	1,165,476 (18.1)	51,358 (17.3)
≥3	786,727 (12.2)	37,387 (12.6)
**Season of Conception**		
Winter	1,686,752 (25.8)	77,603 (25.5)
Spring	1,634,191 (25.0)	75,312 (24.7)
Summer	1,565,866 (23.9)	74,181 (24.4)
Fall	1,642,220 (25.1)	76,733 (25.2)

**Table 2:** Adjusted prevalence ratios and 95% confidence intervals for the associations between replicated birth defects and season of conception, estimated among all study subjects.

BPA4 Code	Birth Defect Name	Season of Conception	PR (95% CI) ^1^
	Any monitored birth defect	Spring	1.00 (0.99, 1.01)
		Summer	1.03 (1.02, 1.04)
		Fall	1.02 (1.01. 1.03)
742.2	Reduction anomalies of brain	Spring	1.07 (0.99, 1.15)
		Summer	1.14 (1.05, 1.23)
		Fall	1.05 (0.98, 1.14)
750.5	Congenital hypertrophic pyloric stenosis	Spring	0.89 (0.84, 0.94)
		Summer	1.02 (0.96, 1.07)
		Fall	1.02 (0.97, 1.08)
751.3	Hirschsprung’s disease and other congenital functional disorders of the colon	Spring	1.03 (0.85, 1.24)
		Summer	1.46 (1.22, 1.75)
		Fall	1.06 (0.88, 1.28)
754.5	Varus (inward) deformities of feet	Spring	1.04 (0.97, 1.11)
		Summer	1.14 (1.06, 1.22)
		Fall	1.03 (0.96, 1.11)
755.6	Other anomalies of lower limb, including pelvic girdle	Spring	1.04 (0.99, 1.09)
		Summer	1.10 (1.04, 1.15)
		Fall	1.04 (0.99, 1.09)
756.0	Anomalies of skull and face bones	Spring	1.04 (0.99, 1.08)
		Summer	1.10 (1.05, 1.15)
		Fall	1.05 (1.01, 1.10)

PR = prevalence ratio. ^1^ Adjusted for maternal age, race/ethnicity, education, and number of previous livebirths.

## Supplementary Materials

The following are available online at https://www.mdpi.com/1660-4601/17/19/7120/s1, Table S1: Adjusted prevalence ratios and 95% confidence intervals for birth defects associated with season of conception (discovery partition), Table S2: Adjusted prevalence ratios and 95% confidence intervals for birth defects associated with season of conception (replication partition).

## References

[1] Centers for Disease Control and Prevention (CDC) Data and Statistics on Birth Defects. https://www.cdc.gov/ncbddd/birthdefects/data.html.

[2] Centers for Disease Control and Prevention (CDC) What are Birth Defects?. https://www.cdc.gov/ncbddd/birthdefects/facts.html.

[3] Stanford Children’s Health Birth Defects in Children. https://www.stanfordchildrens.org/en/topic/default?id=overview-of-birth-defects-90-P02113.

[4] United States Environmental Protection Agency (EPA) Supplementary Topics: Birth Defects. https://www.epa.gov/sites/production/files/2015-06/documents/supplementary-topics-birth-defects.pdf.

[5] Øyen N., Diaz L.J., Leirgul E., Boyd H.A., Priest J., Mathiesen E.R., Quertermous T., Wohlfahrt J., Melbye M. (2016). Prepregnancy diabetes and offspring risk of congenital heart disease: A nationwide cohort study. Circulation.

[6] Siebold B., Heike C.L., Leroux B.G., Speltz M.L., Drake A.F., Johns A.L., Kapp-Simon K.A., Magee L., Luquetti D.V. (2019). Evaluation of prenatal diabetes mellitus and other risk factors for craniofacial microsomia. Birth Defects Res..

[7] Vinceti M., Malagoli C., Rothman K.J., Rodolfi R., Astolfi G., Calzolari E., Puccini A., Bertolotti M., Lunt M., Paterlini L. (2014). Risk of birth defects associated with maternal pregestational diabetes. Eur. J. Epidemiol..

[8] Tomson T., Battino D., Perucca E. (2019). Teratogenicity of Antiepileptic Drugs. Curr. Opin. Neurol..

[9] Kashif T., Fathima N., Usman N., Qaseem A., Jayaraj J.S. (2019). Women with epilepsy: Anti-epileptic drugs and perinatal outcomes. Cureus.

[10] Caton A.R. (2012). Exploring the seasonality of birth defects in the New York state congenital malformations registry. Birth Defects Res. Part A-Clin. Mol. Teratol..

[11] Luteijn J.M., Dolk H., Addor M.C., Arriola L., Barisic I., Bianchi F., Calzolari E., Draper E., Garne E., Gatt M. (2014). Seasonality of congenital anomalies in Europe. Birth Defects Res. Part A-Clin. Mol. Teratol..

[12] Peterka M., Likovsky Z., Panczak A., Peterkova R. (2018). Long-term significant seasonal differences in the numbers of new-borns with an orofacial cleft in the Czech Republic—A retrospective study. BMC Pregnancy Childbirth.

[13] Centers for Disease Control and Prevention (CDC) State-Based Birth Defects Tracking Systems. https://www.cdc.gov/ncbddd/birthdefects/states/index.html.

[14] Roden D.M. (2017). Phenome-wide association studies: A new method for functional genomics in humans. J. Physiol..

[15] Hebbring S.J. (2014). The challenges, advantages and future of phenome-wide association studies. Immunology.

[16] Schraw J.M., Langlois P.H., Lupo P.J. (2020). Comprehensize assessment of the assoications between maternal diabetes and structural birth defects in offspring: A phenome-wide association study. Ann. Epidemiol..

[17] von Elm E., Altman D.G., Egger M., Pocock S.J., Gøtzsche P.C., Vandenbroucke J.P. (2014). The strengthening the reporting of observational studies in Epidemiology (STROBE) statement: Guidelines for reporting observational atudies. Int. J. Surg..

[18] National Centers for Environmental Information (NCEI), National Oceanic and Atmospheric Administration (NOAA) Meteorological Versus Astronomical Seasons. https://www.ncei.noaa.gov/news/meteorological-versus-astronomical-seasons.

[19] Zhang W., Spero T.L., Nolte C.G., Garcia V.C., Lin Z., Romitti P.A., Shaw G.M., Sheridan S.C., Feldkamp M.L., Woomert A. (2019). Projected changes in maternal heat exposure during early pregnancy and the associated congenital heart defect burden in the United States. J. Am. Heart Assoc..

[20] Lin S., Lin Z., Ou Y., Soim A., Shrestha S., Lu Y., Sheridan S., Luben T.J., Fitzgerald E., Bell E. (2018). Maternal ambient heat exposure during early pregnancy in summer and spring and congenital heart defects—A large US population-based, case-control study. Environ. Int..

[21] De la Vega A., Martinez E. (2006). Seasonal variation in the incidence of cleft lip and palate based on the age of conception. Puerto Rico Health Sci. J..

[22] Siffel C., Alverson C.J., Correa A. (2005). Analysis of seasonal variation of birth defects in Atlanta. Birth Defects Res. Part A Clin. Mol. Teratol..

[23] Rogerson P.A. (1996). A generalization of Hewitt’s Test for seasonality. Int. J. Epidemiol..

[24] Walter S.D., Elwood J.M. (1975). A test for seasonality of events with a variable population at risk. J. Epidemiol. Community Health.

[25] Weather Atlas Texas, USA—Climate Data and Average Monthly Weather. https://www.weather-us.com/en/texas-usa-climate#climate_text_1.

[26] Stingone J.A., Luben T.J., Sheridan S.C., Langlois P.H., Shaw G.M., Reefhuis J., Romitti P.A., Feldkamp M.L., Nembhard W.N., Browne M.L. (2019). Associations between fine particulate matter, extreme heat events, and congenital heart defects. Environ. Epidemiol..

[27] Bennett G.D. (2010). Hyperthermia: Malformations to chaperones. Birth Defects Res. Part B Dev. Reprod. Toxicol..

[28] Bekkar B., Pacheco S., Basu R., DeNicola N. (2020). Association of air pollution and heat exposure with preterm birth, low birth weight, and stillbirth in the US. JAMA Netw. Open.

[29] Kirkbride J.B., Susser E., Kundakovic M., Kresovich J.K., Davey Smith G., Relton C.L. (2012). Prenatal nutrition, epigenetics and schizophrenia risk: Can we test causal effects?. Epigenomics.

[30] Verdoux H., Takei N., De Saint-Mathurin R.C., Bourgeois M. (1997). Analysis of the seasonal variation of schizophrenic births using a Kolmogorov-Smirnov Type statistic. Eur. Psychiatry.

[31] Dowell S.F. (2001). Seasonal variation in host susceptibility and cycles of certain infectious diseases. Emerg. Infect. Dis..

