# Supplementary material for: A Comprehensive Assessment of the Associations Between Season of Conception and Birth Defects, Texas, 1999–2015
Source: Int J Environ Res Public Health. 2020 Sep 29;17(19):7120. doi: 10.3390/ijerph17197120 (PMC7579376; doi:10.3390/ijerph17197120)
Supplement: Supplementary file 1 [file ijerph-17-07120-s001.pdf]

Table S1. Adjusted prevalence ratios and 95% confidence intervals for birth defects associated with season of conception (discovery partition).

BPA4 code	Birth defect name	Season of Conception	PR (95% CI)[1]
742.1	Microcephaly	Spring	1.07 (0.99, 1.16)
		Summer	1.10 (1.01, 1.19)
		Fall	1.06 (0.98, 1.15)
742.2	Reduction anomalies of brain	Spring	1.08 (0.98, 1.19)
		Summer	1.13 (1.03, 1.25)
		Fall	1.08 (0.98, 1.19)
742.4	Other specified brain anomalies	Spring	1.00 (0.93, 1.06)
		Summer	1.08 (1.01, 1.15)
		Fall	1.02 (0.96, 1.09)
742.8	Other specified anomalies of nervous system	Spring	1.49 (1.05, 2.09)
		Summer	0.88 (0.59, 1.3)
		Fall	1.14 (0.79, 1.64)
743.2	Buphthalmos	Spring	1.50 (1.06, 2.13)
		Summer	1.08 (0.74, 1.58)
		Fall	1.32 (0.92, 1.89)
743.6	Eyelid, lacrimal, orbit anomalies	Spring	1.02 (0.92, 1.13)
		Summer	1.09 (0.98, 1.20)
		Fall	1.13 (1.03, 1.25)
743.8	Other specified anomalies of eye	Spring	0.44 (0.19, 1.00)
		Summer	0.90 (0.46, 1.75)
		Fall	0.70 (0.34, 1.41)
744.0	Anomalies of ear causing impairment of hearing	Spring	1.05 (0.92, 1.21)
		Summer	1.16 (1.01, 1.32)
		Fall	1.11 (0.97, 1.27)
744.2	Other specified anomalies of ear	Spring	1.03 (0.98, 1.09)
		Summer	1.07 (1.02, 1.13)
		Fall	0.98 (0.93, 1.04)
744.3	Unspecified anomalies of ear	Spring	1.16 (0.86, 1.57)
		Summer	0.66 (0.46, 0.94)
		Fall	0.83 (0.60, 1.15)
744.4	Branchial cleft, cyst, or fistula; preauricular sinus	Spring	1.12 (0.88, 1.41)
		Summer	1.38 (1.10, 1.73)
		Fall	1.00 (0.79, 1.28)
745.0	Common truncus	Spring	0.79 (0.59, 1.06)
		Summer	0.74 (0.55, 1.00)

		Fall	0.65 (0.48, 0.88)
745.5	Ostium secundum type atrial septal defect	Spring	1.00 (0.97, 1.02)
		Summer	1.02 (1.00, 1.05)
		Fall	1.03 (1.01, 1.06)
747.6	Endocardial cushion defects	Spring	1.03 (0.88, 1.21)
		Summer	1.17 (1.00, 1.37)
		Fall	1.09 (0.93, 1.28)
748.4	Congenital cystic lung	Spring	1.20 (0.88, 1.64)
		Summer	1.38 (1.02, 1.87)
		Fall	1.23 (0.91, 1.68)
750.5	Congenital hypertrophic pyloric stenosis	Spring	0.91 (0.85, 0.98)
		Summer	1.03 (0.97, 1.11)
		Fall	1.04 (0.97, 1.11)
750.7	Other specified anomalies of stomach	Spring	0.98 (0.71, 1.34)
		Summer	0.76 (0.54, 1.07)
		Fall	0.61 (0.42, 0.87)
751.3	Hirschsprung's disease and other congenital functional disorders of the colon	Spring	1.05 (0.82, 1.35)
		Summer	1.49 (1.18, 1.87)
		Fall	1.04 (0.81, 1.33)
751.5	Other anomalies of intestine	Spring	1.15 (1.00, 1.32)
		Summer	1.16 (1.01, 1.33)
		Fall	1.23 (1.08, 1.41)
752.7	Indeterminate sex and pseudohermaphroditism	Spring	1.35 (1.00, 1.82)
		Summer	1.10 (0.79, 1.51)
		Fall	1.36 (1.01, 1.84)
753.0	Renal agenesis and dysgenesis	Spring	1.07 (0.96, 1.20)
		Summer	1.13 (1.01, 1.27)
		Fall	1.00 (0.89, 1.13)
753.1	Cystic kidney disease	Spring	0.92 (0.83, 1.03)
		Summer	0.97 (0.88, 1.08)
		Fall	0.87 (0.78, 0.97)
753.3	Other specified anomalies of kidney	Spring	1.13 (1.01, 1.26)
		Summer	1.15 (1.03, 1.29)
		Fall	1.00 (0.90, 1.12)
754.5	Varus (inward) deformities of feet	Spring	1.01 (0.93, 1.11)
		Summer	1.15 (1.06, 1.25)
		Fall	1.06 (0.97, 1.16)

755.6	Other anomalies of lower limb, including pelvic girdle	Spring	1.05 (0.99, 1.12)
		Summer	1.09 (1.02, 1.16)
		Fall	1.02 (0.95, 1.08)
756.0	Anomalies of skull and face bones	Spring	1.00 (0.94, 1.05)
		Summer	1.09 (1.03, 1.15)
		Fall	1.05 (0.99, 1.10)
756.3	Other anomalies of ribs and sternum	Spring	1.06 (0.92, 1.21)
		Summer	1.25 (1.09, 1.42)
		Fall	1.05 (0.91, 1.20)
756.8	Other specified anomalies of muscle, tendon, fascia and connective tissue	Spring	1.08 (1.02, 1.16)
		Summer	1.06 (0.99, 1.14)
		Fall	1.04 (0.97, 1.11)
757.5	Specified anomalies of nails	Spring	0.93 (0.80, 1.08)
		Summer	0.95 (0.82, 1.10)
		Fall	0.82 (0.72, 0.98)
758.8	Other conditions due to sex chromosome anomalies	Spring	1.23 (0.88, 1.71)
		Summer	1.18 (0.85, 1.66)
		Fall	1.45 (1.05, 1.99)
758.9	Other conditions due to anomaly of unspecified chromosomes	Spring	3.01 (1.56, 5.80)
		Summer	2.07 (1.03, 4.16)
		Fall	1.63 (0.79, 3.37)
759.3	Situs inversus	Spring	3.01 (1.56, 5.80)
		Summer	2.07 (1.03, 4.16)
		Fall	1.63 (0.79, 3.37)
759.6	Other hamartoses, not elsewhere classified	Spring	1.70 (1.13, 2.56)
		Summer	1.50 (0.99, 2.29)
		Fall	1.25 (0.81, 1.92)
759.7	Multiple congenital anomalies	Spring	0.78 (0.53, 1.14)
		Summer	0.85 (0.58, 1.24)
		Fall	0.61 (0.40, 0.92)
888.8	Any monitored birth defect	Spring	1.00 (0.99, 1.01)
		Summer	1.03 (1.02, 1.05)
		Fall	1.02 (1.00, 1.03)

PR = prevalence ratio

1. Adjusted for maternal age, race/ethnicity, education, and number of previous livebirths.

Table S2. Adjusted prevalence ratios and 95% confidence intervals for birth defects associated with season of conception (replication partition).

BPA4 code	Birth defect name	Season of Conception	PR (95% CI)[1]
742.1	Microcephaly	Spring	1.06 (0.96, 1.18)
		Summer	1.10 (0.99, 1.21)
		Fall	1.08 (0.97 1.19)
742.2	Reduction anomalies of brain	Spring	1.05 (0.93, 1.18)
		Summer	1.14 (1.01, 1.28)
		Fall	1.01 (0.89, 1.14)
742.4	Other specified brain anomalies	Spring	0.93 (0.85 1.00)
		Summer	1.02 (0.94, 1.10)
		Fall	0.96 (0.88, 1.04)
742.8	Other specified anomalies of nervous system	Spring	1.07 (0.70, 1.62)
		Summer	0.92 (0.59, 1.43)
		Fall	0.86 (0.55, 1.34)
743.2	Buphthalmos	Spring	0.89 (0.58, 1.37)
		Summer	0.93 (0.61, 1.43)
		Fall	0.98 (0.65, 1.49)
743.6	Eyelid, lacrimal, orbit anomalies	Spring	1.04 (0.92, 1.18)
		Summer	1.02 (0.90, 1.15)
		Fall	1.00 (0.88, 1.13)
743.8	Other specified anomalies of eye	Spring	1.03 (0.41, 2.61)
		Summer	1.09 (0.43, 2.74)
		Fall	1.15 (0.47, 2.82)
744.0	Anomalies of ear causing impairment of hearing	Spring	0.99 (0.84, 1.17)
		Summer	1.06 (0.90, 1.25)
		Fall	1.16 (0.99, 1.36)
744.2	Other specified anomalies of ear	Spring	0.99 (0.93, 1.06)
		Summer	1.01 (0.94, 1.08)
		Fall	1.03 (0.96, 1.10)
744.3	Unspecified anomalies of ear	Spring	0.96 (0.64, 1.43)
		Summer	1.12 (0.75, 1.65)
		Fall	0.89 (0.59 1.34)
744.4	Branchial cleft, cyst, or fistula; preauricular sinus	Spring	0.71 (0.53, 0.96)
		Summer	0.96 (0.73, 1.27)
		Fall	1.03 (0.78, 1.34)
745.0	Common truncus	Spring	1.25 (0.84, 1.89)
		Summer	1.70 (1.16, 2.49)

		Fall	1.37 (0.92, 2.03)
745.5	Ostium secundum type atrial septal defect	Spring	0.99 (0.96, 1.02)
		Summer	1.01 (0.98, 1.04)
		Fall	1.00 (0.97, 1.03)
747.6	Other anomalies of peripheral vascular system	Spring	0.98 (0.81, 1.18)
		Summer	0.98(0.81, 1.18)
		Fall	0.84 (0.69, 1.02)
748.4	Congenital cystic lung	Spring	1.02 (0.70, 1.47)
		Summer	0.94 (0.64, 1.37)
		Fall	1.20 (0.85, 1.72)
750.5	Congenital hypertrophic pyloric stenosis	Spring	0.86 (0.79, 0.94)
		Summer	0.99 (0.91, 1.07)
		Fall	1.00 (0.92, 1.08)
750.7	Other specified anomalies of stomach	Spring	0.87 (0.57, 1.34)
		Summer	0.85 (0.55 1.32)
		Fall	1.12 (0.75, 1.67)
751.3	Hirschsprung's disease and other congenital functional disorders of the colon	Spring	0.99 (0.73, 1.34)
		Summer	1.42 (1.07, 1.89)
		Fall	1.09 (0.81, 1.47)
751.5	Other anomalies of intestine	Spring	0.94 (0.80, 1.11)
		Summer	1.00 (0.85 1.17)
		Fall	0.87 (0.73 1.02)
752.7	Indeterminate sex and pseudohermaphroditism	Spring	0.76 (0.53, 1.07)
		Summer	0.85 (0.61, 1.20)
		Fall	0.72 (0.50, 1.02)
753.0	Renal agenesis and dysgenesis	Spring	0.93 (0.81, 1.07)
		Summer	0.97 (0.84, 1.11)
		Fall	1.01 (0.89, 1.16)
753.1	Cystic kidney disease	Spring	0.97 (0.85, 1.11)
		Summer	1.09 (0.95, 1.24)
		Fall	0.98 (0.86, 1.12)
753.3	Other specified anomalies of kidney	Spring	0.94 (0.82, 1.07)
		Summer	1.05 (0.92, 1.19)
		Fall	0.89 (0.78, 1.02)
754.5	Varus (inward) deformities of feet	Spring	1.07 (0.96, 1.19)
		Summer	1.12 (1.00, 1.24)
		Fall	0.99 (0.88, 1.10)
755.6	Other anomalies of lower limb, including pelvic girdle	Spring	1.02 (0.94, 1.10)

		Summer	1.10 (1.02, 1.20)
		Fall	1.06 (0.98, 1.15)
756.0	Anomalies of skull and face bones	Spring	1.10 (1.03, 1.18)
		Summer	1.11 (1.04, 1.19)
		Fall	1.05 (0.99, 1.13)
756.3	Other anomalies of ribs and sternum	Spring	0.95 (0.81, 1.12)
		Summer	1.07 (0.91, 1.26)
		Fall	0.98 (0.83, 1.15)
756.8	Other specified anomalies of muscle, tendon, fascia and connective tissue	Spring	1.01 (0.92, 1.09)
		Summer	1.00 (0.92, 1.09)
		Fall	1.01 (0.93, 1.10)
757.5	Specified anomalies of nails	Spring	1.01 (0.84, 1.22)
		Summer	1.13 (0.94, 1.36)
		Fall	0.99 (0.82, 1.19)
758.8	Other conditions due to sex chromosome anomalies	Spring	0.95 (0.65,1.37)
		Summer	0.88 (0.60, 1.29)
		Fall	0.78 (0.53, 1.16)
758.9	Other conditions due to anomaly of unspecified chromosomes	Spring	0.62 (0.30, 1.28)
		Summer	0.71 (0.35, 1.42)
		Fall	0.73 (0.37, 1.44)
759.3	Situs inversus	Spring	1.01 (0.77, 1.33)
		Summer	0.90 (0.68, 1.19)
		Fall	0.87 (0.66, 1.15)
759.6	Other hamartoses, not elsewhere classified	Spring	1.01 (0.63, 1.61)
		Summer	0.84 (0.51, 1.37)
		Fall	0.86 (0.53, 1.39)
759.7	Multiple congenital anomalies	Spring	0.98 (0.62, 1.56)
		Summer	0.82 (0.50, 1.34)
		Fall	0.70 (0.42, 1.16)
888.8	Any monitored birth defect	Spring	1.00 (0.99, 1.02)
		Summer	1.02 (1.01, 1.04)
		Fall	1.02 (1.00, 1.03)

PR = prevalence ratio

1. Adjusted for maternal age, race/ethnicity, education, and number of previous livebirths.
